# Effectiveness of low-Dye taping for the short-term treatment of plantar heel pain: a randomised trial

**DOI:** 10.1186/1471-2474-7-64

**Published:** 2006-08-09

**Authors:** Joel A Radford, Karl B Landorf, Rachelle Buchbinder, Catherine Cook

**Affiliations:** 1 School of Biomedical and Health Sciences, University of Western Sydney, Locked Bag 1797, Penrith South DC, NSW, 1797, Australia; 2 Department of Podiatry, School of Human Biosciences, La Trobe University, Bundoora, Victoria, 3086 Australia; 3 Monash Department of Clinical Epidemiology at Cabrini Hospital and Department of Epidemiology and Preventive Medicine, Monash University, Malvern, Victoria, 3144, Australia

## Abstract

**Background:**

Plantar heel pain is one of the most common musculoskeletal disorders of the foot and ankle. Treatment of the condition is usually conservative, however the effectiveness of many treatments frequently used in clinical practice, including supportive taping of the foot, has not been established. We performed a participant-blinded randomised trial to assess the effectiveness of low-Dye taping, a commonly used short-term treatment for plantar heel pain.

**Methods:**

Ninety-two participants with plantar heel pain (mean age 50 ± 14 years; mean body mass index 30 ± 6; and median self-reported duration of symptoms 10 months, range of 2 to 240 months) were recruited from the general public between February and June 2005. Participants were randomly allocated to (i) low-Dye taping and sham ultrasound or (ii) sham ultrasound alone. The duration of follow-up for each participant was one week. No participants were lost to follow-up. Outcome measures included 'first-step' pain (measured on a 100 mm Visual Analogue Scale) and the Foot Health Status Questionnaire domains of foot pain, foot function and general foot health.

**Results:**

Participants treated with low-Dye taping reported a small improvement in 'first-step' pain after one week of treatment compared to those who did not receive taping. The estimate of effect on 'first-step' pain favoured the low-Dye tape (ANCOVA adjusted mean difference -12.3 mm; 95% CI -22.4 to -2.2; *P *= 0.017). There were no other statistically significant differences between groups. Thirteen participants in the taping group experienced an adverse event however most were mild to moderate and short-lived.

**Conclusion:**

When used for the short-term treatment of plantar heel pain, low-Dye taping provides a small improvement in 'first-step' pain compared with a sham intervention after a one-week period.

## Background

Plantar heel pain (plantar fasciitis) can be a painful and debilitating condition. It is a common disorder in both athletic [1] and sedentary populations [2-4]. A recent United States study estimated that each year one million patient visits at office-based physicians and hospital outpatient departments are for the diagnosis and treatment of heel pain[5]. Seven percent of adults aged 65 years or older have been found to have plantar heel pain[2,3]. The disorder is thought to be multifactorial in origin with factors such as obesity, excessive periods of weightbearing activity and decreased ankle range of motion commonly suggested to be involved [6].

A wide variety of management strategies have been developed to treat the disorder. A systematic review [7] identified 26 different conservative treatments that have been recommended for the treatment of plantar heel pain. Only heel pads, orthoses, steroid injections, night splints and extracorporeal shock wave therapy have been evaluated in randomised trials.

Foot orthoses are a common treatment for plantar heel pain, however due to the manufacturing process, they often require a period of a few weeks between the initial consultation and issuing the devices. As such, short-term treatments such as supportive taping are used to alleviate symptoms during this interim period[8] – the low-Dye [9] taping technique being one of the most frequently used. A systematic review[10] of randomised crossover trials examined the effect of low-Dye taping on biomechanical variables. Like foot orthoses, it is thought that supportive tape reduces the symptoms of plantar heel pain by reducing strain in the plantar fascia during standing and ambulation [11]. A reduction in strain is achieved by reducing navicular drop upon weightbearing (i.e. arch collapse)[10]. However, it is unknown if a change in a surrogate outcome measure such as navicular drop actually correlates to an improvement for the patient.

To this date, the effect of low-Dye taping on patient outcomes has only been assessed in non-randomised trials with beneficial results reported[12,13]. We therefore conducted a participant-blinded randomised trial to determine whether low-Dye taping is an effective short-term treatment for plantar heel pain.

## Methods

A randomised, participant-blinded trial was conducted between February and June 2005. Participants with a clinical diagnosis of plantar heel pain and who provided informed consent were randomly allocated to one of two groups: (i) low-Dye taping with sham ultrasound, or (ii) sham ultrasound only. Participants were informed prior to entering the study that a sham intervention was being administered in the trial and were blinded as to whether they were receiving active treatment or not. Ethical approval for the trial was gained from the University of Western Sydney Human Research Ethics Committee. The trial was registered with the Australian Clinical Trials Registry (ACTRN012605000046606) which meets the requirements of the International Committee of Medical Journal Editors (ICMJE) for trial registration [14].

### Participants

Participants were included if diagnosed with plantar heel pain described as (i) a localised pain at the plantar heel; (ii) that was worst when first standing or walking after rest; and (iii) that improved initially after first standing but worsened with increasing activity. Participants also needed to be ≥ 18 years of age and have had experienced symptoms for ≥ four weeks. Participants were excluded from the trial if they had any inflammatory, osseous, metabolic or neurological abnormalities or had received a corticosteroid injection within the past three months. Participants with known tape allergies were also excluded. Participants were encouraged not to commence use of any new treatments during the trial (e.g. anti-inflammatory medication, foot orthoses etc.).

### Clinical protocol

Participants were recruited from local community newspaper advertisements in Campbelltown (Sydney, Australia) and treated at a university podiatry clinic. The random allocation sequence was generated using a computer program (Microsoft Excel) in one block (i.e. simple randomisation). The allocation sequence was concealed from the researcher (JR) enrolling and assessing participants in sequentially numbered, opaque, sealed and stapled envelopes. Aluminium foil inside the envelope was used to render the envelope impermeable to intense light. To prevent subversion of the allocation sequence, the name and date of birth of the participant was written on the envelope and a video tape made of the sealed envelope with participant details visible. Carbon paper inside the envelope transferred the information onto the allocation card inside the envelope and a second researcher (CC) later viewed video tapes to ensure envelopes were still sealed when participants' names were written on them. Corresponding envelopes were opened only after the enrolled participants completed all baseline assessments and it was time to allocate the intervention.

Three minutes of sham ultrasound (Accusonic AS250, Metron) was then given to the painful heel regardless of whether participants had been allocated the active intervention (i.e. tape) or not. The ultrasound unit was powered with all operational lights activated. At commencement of treatment the researcher (JR) turned up the wattage of the machine which was accompanied by 'beeping' sounds. However, no ultrasound was emitted as the internal timer was not activated; an external timer was used instead.

**Figure 1:**
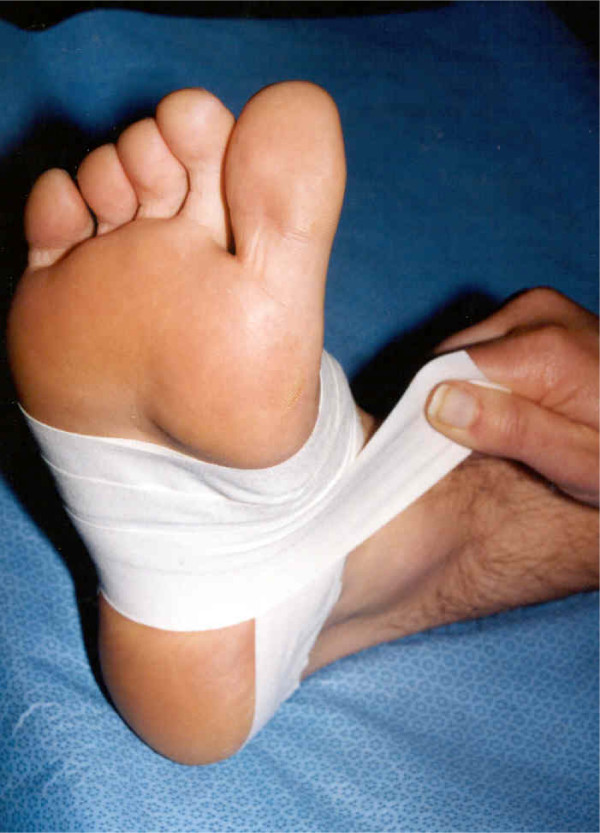
Low-Dye taping.

Participants in the taping group were then taped using the low-Dye taping technique[13] (participants allocated the sham treatment did not receive tape). The skin was first swabbed with alcohol and then Leuko^® ^Spray Adhesive (Beiersdorf Australasia Ltd) was sprayed on the foot to assist tape adhesion. Low-Dye taping was then sequentially applied using Leuko^® ^Premium Plus (flesh coloured) Sports Tape 3.8 cm wide (Beiersdorf Australasia Ltd.). The technique used (Figure 1) has been described in detail elsewhere[13]. Tape required for one application cost approximately AU$1.00. In the absence of adverse events, participants allocated to taping were instructed to leave the tape on until their follow-up appointment (one week later). Care was taken that study participants did not meet by ensuring they exited the building by a different doorway to the one through which they entered.

Outcome assessment was performed at baseline and at one week. Baseline variables that were collected included age, sex, weight, self-reported hours on feet, duration of symptoms, foot posture, ankle range of motion. Outcome measures included: (i) 'first-step' pain – the pain experienced when first standing after arising from bed in the morning, measured on a 100 mm Visual Analogue Scale; and (ii) the Foot Health Status Questionnaire which has four domains covering foot pain, foot function, footwear and general foot health (although we pre-specified that we would not analyse the footwear domain). The Foot Health Status Questionnaire – a self-rated health status measure – has been previously validated (content, criterion and construct validity) across a wide spectrum of pathologies including skin, nail and musculoskeletal disorders. It has a high test-retest reliability (intraclass correlation coefficients ranging from 0.74 to 0.92) and a high degree of internal consistency (Cronbach's α ranging from 0.85 to 0.88)[15]. To minimise the investigator having influence on participant responses, participants completed the outcome assessments prior to each consultation.

Participants with bilateral symptoms only received treatment for the foot with the worst symptoms and participants were asked to concentrate on the pain in that foot when completing all outcome measures. Participants who removed tape before returning were asked to concentrate on the symptoms they had experienced while the tape was on the foot. The number of days participants wore the tape was recorded.

### Sample size, data handling and analysis

The sample size of 92 (i.e. 46 per group) was calculated *a priori*. This sample size provides an 80% probability of detecting a minimal important difference [16] of 14 points between the groups on the pain domain of the Foot Health Status Questionnaire (standard deviation 24 and alpha level 0.05). This sample size also provided adequate power to detect a minimal important difference of 9 points on the Visual Analogue Scale (standard deviation 15.5). We conservatively ignored the extra precision provided by covariate analysis when estimating sample size.

Outcome data were analysed by intention to treat and according to a pre-planned protocol (i.e. *a priori*). To maximise precision of estimates, analysis of covariance (ANCOVA) was conducted using a linear regression approach[17,18]. The outcomes analysed were the change in 'first-step' pain (Visual Analogue Scale) and the change in foot pain, foot function and general foot health (Foot Health Status Questionnaire). We pre-specified that the *baseline outcome measure *(i.e. 'first-step' pain or Foot Health Status Questionnaire data at baseline) would be used as the only covariate in each analysis[19]. For the analysis of 'first-step' pain we adjusted for 'first-step' pain at baseline. For the analysis of Foot Health Status Questionnaire foot pain, foot function and general foot health we adjusted for these domains at baseline. Independent researchers (not otherwise involved in the trial – see acknowledgments) performed data entry and were blinded to group allocation. Double data entry was used to check for errors. Statistical analyses were performed while researchers were blinded to group allocation.

An independent sample t-test was used to determine if there was a difference between groups in the number of days between baseline and follow-up appointments. Participants were also asked which intervention (active or sham) they thought they had received and an index[20] calculated to assess the success of blinding. The index takes the value 1 for complete blinding and 0 for complete lack of blinding; 0.5 is the equivalent of random guessing.

## Results

Baseline characteristics (Table 1) between groups were similar in all outcomes, although the taping group had a higher percentage of bilateral cases than the sham group (63% to 48%). Participants were primarily middle-aged (mean 50 years; SD ± 14), female (55 women; 60%), overweight (BMI mean 30; SD ± 6); spent the majority of the day on their feet (mean 8 hours; SD ± 3) and presented with relatively chronic pain (median 10 months, range 2 to 240).

**Figure 2:**
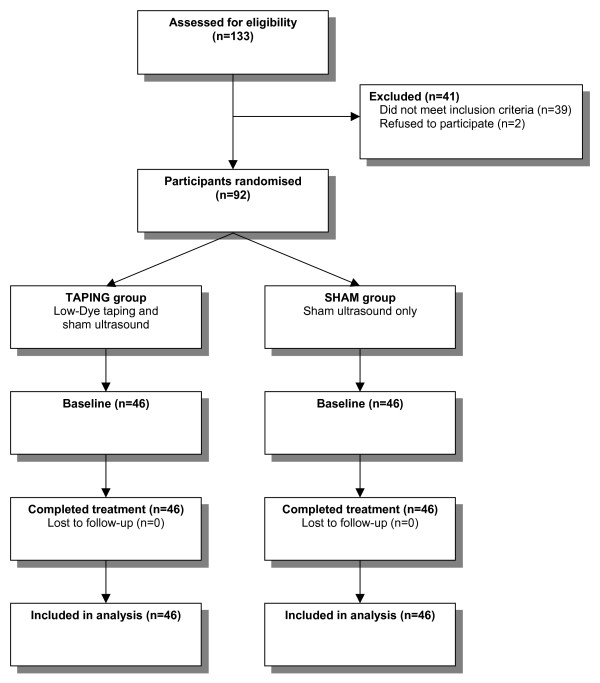
Participant flow diagram.

Figure 2 displays the flow of participants through the trial. There were no participants lost to follow-up. There was no difference between the groups in time to follow-up (p = 0.378). From baseline to the review appointment the mean number of days was 7.2 days (SD ± 0.7) for the taping group and 7.2 days (SD ± 0.6) for the placebo group. Participants in the taping group left the tape on for a median of 7 days (range 3 to 9 days).

**Figure 3:**
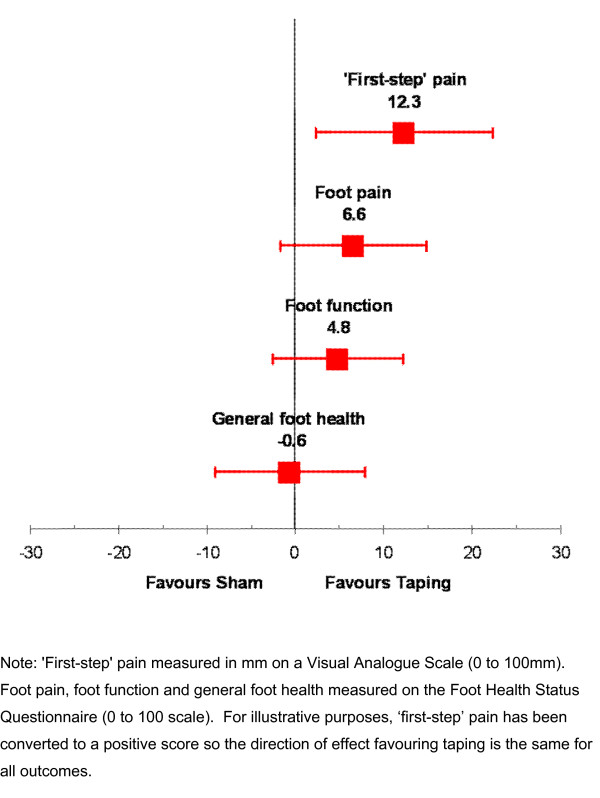
Forest plot of adjusted mean differences with 95% confidence intervals for outcomes.

Both the taping and sham groups improved in 'first-step' pain, foot pain and foot function at follow-up (Table 2). Only for 'first-step' pain did the taping group experience a greater improvement compared to the sham group (ANCOVA adjusted mean difference -12.3 mm; 95% confidence interval -22.4 to -2.2). This difference was statistically significant (*P *= 0.017). Although there were small differences between groups with respect to improvements in the Foot Health Status Questionnaire none were statistically significant (Table 2). These results are presented graphically in Figure 3.

In the taping group, 13 participants (28%) experienced adverse events while no events were reported by the sham group. Adverse events were described as taping too tight (n = 4), a new pain in lower limb (n = 5) and allergic reaction to the tape (n = 4). Adverse events were recorded as mild (n = 5), moderate (n = 5) or severe (n = 3) in nature. Five participants removed their tape before their final appointment due to adverse events (three due to allergic reactions and two due to tightness of the tape). Upon tape removal, all adverse events resolved.

With respect to blinding, thirty-eight participants (83%) in the taping group correctly identified their treatment group compared with three participants (7%) in the sham group. Five participants (11%) in the taping group were uncertain which treatment they received, compared with twenty participants (43%) in the sham group and three participants (7%) the taping group and twenty-three participants (50%) in the sham group incorrectly identified their treatment group. The index to assess the success of blinding[20] was computed to be 0.61 (bootstrap 95% confidence interval 0.54 to 0.69). The value of 0.61 represents a statistically significant amount of blinding beyond that expected by chance and demonstrates an acceptable level of blinding for a trial using sham ultrasound.

## Discussion

The results demonstrate that low-Dye taping produces a small but statistically significant beneficial effect on 'first-step' pain compared with sham ultrasound. The difference of 12.3 mm on the 100 mm Visual Analogue Scale is greater than the 9 to 10 mm difference thought to be clinically important [16,21-23]. No significant differences between groups were found for improvements in foot pain, foot function and general foot health as measured using the Foot Health Status Questionnaire at one week. One reason for this trial finding a statistically significant improvement only for 'first-step' pain with taping could be due to it being the symptom of plantar heel pain that is notable to patients. It is well known that this symptom – that is sharp pain when first getting out of bed in the morning – is characteristic of plantar heel pain and for this reason it was one of the inclusion criteria in this trial. Alternatively, the Foot Health Status Questionnaire may not have been sensitive or specific enough to corroborate with the improvement detected in 'first-step' pain (measured explicitly using a 100 mm Visual Analogue Scale).

This is the first randomised trial to evaluate a foot taping technique for plantar heel pain. Two non-randomised trials have previously examined low-Dye taping for plantar heel pain[12,13]. A small case series [12] of only eight participants reported a beneficial effect from the tape although no outcome data were presented. A larger (n = 105) trial[13] reported a 32 mm reduction in pain (95% confidence interval 24 to 40) on a 100 mm Visual Analogue Scale in a taping group compared to a control group; however the intervention was not randomly allocated. The results of the current randomised trial also reflect a 30 mm reduction in pain for the taping group; however the sham group experienced a 19 mm reduction in pain as well. Therefore, our randomised trial suggests that approximately 19 mm of reduction in the non-randomised trial could have been due to confounding factors such as the placebo or Hawthorne effects.

Participant characteristics in this trial were similar to samples in previous heel pain trials [8,13,24,25]. Participants were primarily middle-aged women who were overweight and spent the majority of the day on their feet. Likewise, participants also presented with relatively chronic symptoms.

The majority of adverse events in this trial were described by participants as short-lived and mild to moderate in intensity. Five participants removed the tape prior to follow-up due to an adverse event. Three were due to allergic reactions to the tape and two due to tightness of the tape; all resolved following tape removal. Such minor events could be minimised with the use of hypoallergenic tape and caution in applying tape to reduce tightness.

### Limitations

The findings of this trial need to be viewed in light of some limitations. "Firstly, recruiting volunteers from advertisements in local community newspapers may include participants who are systematically different from those recruited from amongst patients currently seeking care. However, as discussed previously, the characteristics of participants in our trial are similar to participants in other plantar fasciitis trials [8,13,24,25].

Secondly, we only treated the worst foot in bilateral cases to avoid collecting paired data, where outcome data from left and right feet may be highly correlated[26]. Participants were asked to concentrate on the symptoms relating to the treated foot for all outcomes and this may have led to an erroneous estimate of the treatment effect if participants were unable to separate the symptoms from the other unrelated foot. Future trials should consider including only unilateral cases or treat both feet and collect the data from the two feet as one independent sample using outcomes such as the Foot Health Status Questionnaire.

Thirdly, the evidence from this trial is for one particular low-Dye taping technique only. Numerous modifications have been described and future trials may also wish to examine more aggressive taping techniques, such as the technique described by Lange, Chipchase & Evans[27]. These techniques use greater amounts of tape in an attempt to further decrease collapse of the longitudinal arch and subsequent strain on the plantar fascia.

Finally, the trial specifically examined the effect of taping over a one week period as the technique is usually only used as a short-term treatment for plantar heel pain; generally while a patient is awaiting fabrication of a longer-term treatment such as foot orthoses[8]. It would be of interest to evaluate the effectiveness of regular application of the tape over a longer period to investigate whether an extended application has a long-term effect. This may obviate the need to institute more expensive long-term treatments such as foot orthoses; although the risk of a higher incidence of adverse events may not make this worthwhile.

## Conclusion

Low-Dye taping is effective for the short-term treatment of the common symptom of 'first-step' pain in patients with plantar heel pain. The taping technique used in this trial was associated with mild to moderate short-lived adverse events. Low-Dye taping could be used as an inexpensive short-term treatment for plantar heel pain while patients wait for longer-term treatments, such as foot orthoses.

## Competing interests

The author(s) declare that they have no competing interests.

## Authors' contributions

JAR conceptualised the trial, undertook a literature search, designed the trial, collected and analysed the data, interpreted the results and wrote the paper. KBL assisted with the conceptualisation and design of the trial, analysis of the data, interpretation of the results and writing of the paper. RB assisted with the conceptualisation and design of the trial and writing of the paper. CC assisted with the planning of the project and writing of the paper.

## Pre-publication history

The pre-publication history for this paper can be accessed here:



## Figures and Tables

**Table 1:** Baseline characteristics of participants. Values are means (± standard deviation) unless stated otherwise.

**Variable**	**Taping (n = 46)**	**Sham (n = 46)**
Age in years	51.3 (± 13.5)	49.2 (± 13.8)
BMI in kg/m^2^	30.0 (± 4.8)	30.6 (± 6.5)
Self reported hours on feet	8.8 (± 3.8)	7.5 (± 2.8)
Period of symptoms in months – median (range)	9 (3 to 240)	10 (2 to 97)
No. of women (%)	28 (60.9)	27 (58.7)
Foot affected		
Right – No. (%)	10 (21.7)	12 (26.1)
Left – No. (%)	7 (15.2)	12 (26.1)
Both feet – No. (%)	29 (63.0)	22 (47.8)
Foot Posture*		
Right	3.2 (± 2.1)	2.9 (± 2.2)
Left	3.1 (± 2.1)	2.9 (± 2.2)
Ankle range of motion in degrees§		
Right	22.8 (± 7.9)	23.4 (± 6.4)
Left	24.9 (± 7.5)	24.4 (± 6.6)
'First-step' pain†	71.4 (± 19.8)	72.0 (± 20.0)
Foot painΨ	35.5 (± 20.8)	35.8 (± 20.1)
Foot functionΨ	54.3 (± 25.7)	60.6 (± 26.6)
General foot healthΨ	30.7 (± 26.9)	27.0 (± 26.3)

* The Foot Posture Index-6 has a score range of -12 to 12 with negative values for signs of foot supination and positive values for signs of foot pronation. Normal foot posture is reported to be between 0 to 5[28]

§ Ankle range of motion measured using the lunge test[29] a measure of ankle dorsiflexion

† Measured using a Visual Analogue Scale (0 to 100 mm)

Ψ Measured using the Foot Health Status Questionnaire (0 to 100)

**Table 2:** Outcomes at follow-up.

**Outcome measure**	**Group**		**Adjusted mean difference (95% CI)**	**P-value**
			
	**Taping – mean (SD)**	**Sham – mean (SD)**		
'First-step' pain*	41.4 (± 28.5)	54.0 (± 24.8)	-12.3 (-22.4 to -2.2)	0.017
Foot painΨ	60.0 (± 22.0)	53.5 (± 21.0)	6.6 (-1.7 to 14.8)	0.117
Foot functionΨ	72.0 (± 21.9)	70.9 (± 25.0)	4.8 (-2.5 to 12.2)	0.193
General foot healthΨ	43.2 (± 27.7)	41.2 (± 27.4)	-0.6 (-9.1 to 7.9)	0.885

* Measured using a Visual Analogue Scale (0 to 100 mm – 0 is no pain and 100 is the worst pain)

Ψ Measured using the Foot Health Status Questionnaire (0 to 100 – 0 is the worst foot health and 100 is the best foot health)

**Note**: The Foot Health Status Questionnaire is 'reverse-scored' compared to the Visual Analogue Scale. In the results above, the *negative *ANCOVA adjusted mean difference for the comparison of 'first-step' pain favours taping because it has lower pain. In comparison, a *positive *score for the Foot Health Status Questionnaire domains favours the taping group.
